# Phytochemical Analysis and Toxicity Assessment of *Bouea Macrophylla* Yoghurt

**DOI:** 10.3390/toxins15020125

**Published:** 2023-02-03

**Authors:** Rusydatul Nabila Mahmad Rusli, Ruth Naomi, Muhammad Dain Yazid, Hashim Embong, Kokilavani Perumal, Fezah Othman, Azmiza Syawani Jasni, Siti Hadizah Jumidil, Santhra Segaran Balan, Azrina Zainal Abidin, Khairul Kamilah Abdul Kadir, Hasnah Bahari, Zainul Amiruddin Zakaria

**Affiliations:** 1 Department of Human Anatomy, Faculty of Medicine and Health Sciences, Universiti Putra Malaysia, Serdang 43400, Malaysia; 2 Centre for Tissue Engineering and Regenerative Medicine, Faculty of Medicine, Universiti Kebangsaan Malaysia, Kuala Lumpur 56000, Malaysia; 3 Department of Emergency Medicine, Faculty of Medicine, Universiti Kebangsaan Malaysia, Kuala Lumpur 56000, Malaysia; 4 Department of Biomedical Sciences, Faculty of Medicine and Health Sciences, Universiti Putra Malaysia, Serdang 43400, Malaysia; 5 Department of Medical Microbiology and Parasitology, Faculty of Medicine and Health Sciences, Universiti Putra Malaysia, Serdang 43400, Malaysia; 6 Faculty of Health and Life Sciences, Management and Science University, Shah Alam 40100, Malaysia; 7 Bahagian Inovasi dan Komersialisasi (ICD), Institut Penyelidikan Perhutanan Malaysia (FRIM), Kuala Lumpur 52109, Malaysia; 8 Department of Biomedical Sciences, Faculty of Medicine and Health Sciences, Universiti Malaysia Sabah, Kota Kinabalu 88400, Malaysia

**Keywords:** Fruits, *Bouea macrophylla*, phytochemicals, toxicological studies, nutritional studies, animal model

## Abstract

The *Bouea macrophylla* fruit is native to Malaysia and is known for its many beneficial effects on one’s health. Probiotics are well-known for their roles as anti-inflammatory, antioxidant, and anti-tumour properties due to their widespread use. As a result, the purpose of this study was to incorporate the ethanolic extract of *Bouea macrophylla* into yoghurt and then assess the rodents for any toxicological effects. According to the findings of the nutritional analysis, each 100 mL serving of the newly formulated yoghurt contains 3.29 g of fat, 5.79 g of carbohydrates, 2.92 g of total protein, and 2.72 g of sugar. The ability of the newly developed yoghurt to stimulate the growth of Lactobacilli was demonstrated by the fact that the peak intensity of *Lactobacillus* species was measured at 1.2 × 10^6^ CFU/g while the titratable acidity of the lactic acid was measured at 0.599 CFU/g. In order to carry out the toxicological evaluation, forty-eight male Sprague Dawley (SD) rats were utilized. Oral administration of single doses of 2000 mg/kg over the course of 14 days was used for the study of acute toxicity. Subacute toxicity was studied by giving animals *Bouea macrophylla* yoghurt (BMY) at repeated doses of 50, 250, 500, and 1000 mg/kg/day over a period of 28 days, while the control group was given normal saline. The results of the acute toxicity test revealed that rats treated with increasing doses up to a maximum of 2000 mg/kg exhibited no signs of toxicity. After an additional 14 days without treatment, acute toxicity of a single dose (2000 mg/kg) of BMY did not show any treatment-related toxicity in any of the rats that were observed. According to the data from the subacute toxicity study, there were no differences between the treated groups and the control groups in terms of food and water intake, body weight, plasma biochemistry (AST, ALT, ALP, and creatinine), haematological products, or organ weights. The architecture of the liver, heart, and kidney were all found to be normal upon histological examination. This indicates that oral consumption of BMY did not result in any negative effects being manifested in the rodents.

## 1. Introduction

Nowadays, the usage of plants and fruits as alternative supplements has become prominent due to their lesser toxicity and fewer side effects. In addition, the intake of fresh fruits has been shown to play a significant role in reducing the risk of developing chronic diseases, which is a fact that is widely recognised. A tropical fruit tree native to Southeast Asia, *Bouea macrophylla,* is also known as Gandaria mango or Plum mango. It is grown commercially in the ASEAN regions (Malaysia, Thailand, and Indonesia). These plants were reported to have beneficial effects, including the fruit, leaves, and seeds of the fruit. The leaves are commonly consumed raw as a salad in Malaysia and other parts of Asia, whereas the fruits are typically used in the preparation of jam, drinks, and ‘rojak’ [1]. It has been demonstrated that this fruit contains high levels of antioxidant compounds such as flavonoids, polyphenols, vitamin C, and other phytochemical constituents [2]. These mixtures might be answerable for the pharmacological potential of *Bouea macrophylla* as an antioxidant [1], antimicrobial, anticancer [3], antihyperglycemic, and antiphotoaging [4] agent. Consumption of *Bouea macrophylla* fruit can also increase the β-carotene concentration in the blood [5]. However, data on this fruit are still limited and the safety efficacy, particularly in toxicological studies, is still under-explored [5]. 

Recently, probiotics have drawn the attention of researchers due to their anti-inflammatory, antioxidant, and anti-tumour properties [6]. Probiotics are living microorganisms, which, when integrated into yoghurt, are speculated to improve the composition of gut microbiota. In light of the fact that probiotics play an important part in maintaining overall body health and there is a pressing need to find a more value-added and long-term application for *Bouea macrophylla*, the product was therefore developed to combine the positive effects of probiotics found in yoghurt with the pharmacological properties of *Bouea macrophylla*. However, the dosage and safety of consuming natural extracts are not guaranteed, as there is no published data available regarding the toxicity of *Bouea macrophylla* [5], specifically when it is combined with yoghurt. Hence, the purpose of this research is to investigate the acute and subacute oral toxicity of *Bouea macrophylla* yoghurt (BMY) in male Sprague Dawley (SD) rats. 

## 2. Results

### 2.1. Nutritional Analysis of Bouea macrophylla Yoghurt

The nutritional profile of the BMY was expressed as g/100 g of fresh weight without any food preservative, food flavouring and food colourant. Table 1 shows the nutritional profile of BMY formulation.

### 2.2. Microbiological Analysis of Bouea macrophylla Yoghurt

Based on our microbiological analysis, the pH value of BMY during seven days of storage at 4 °C was recorded at 4.51. Our study shows that there were two species of live microorganisms contained in the BMY (as seen in Table 2).

### 2.3. Phytochemical Compound of Ethanolic Boeau macrophylla

The screening and identification of phytochemicals of phenolic compounds and other compounds were conducted under negative ionisation and compounds shown in the Table 3 belong to different groups of phenolic, terpenoid, alkaloid, benzenoid, and other groups of the compound.

### 2.4. Acute Toxicity Assessment

**Figure 1:**
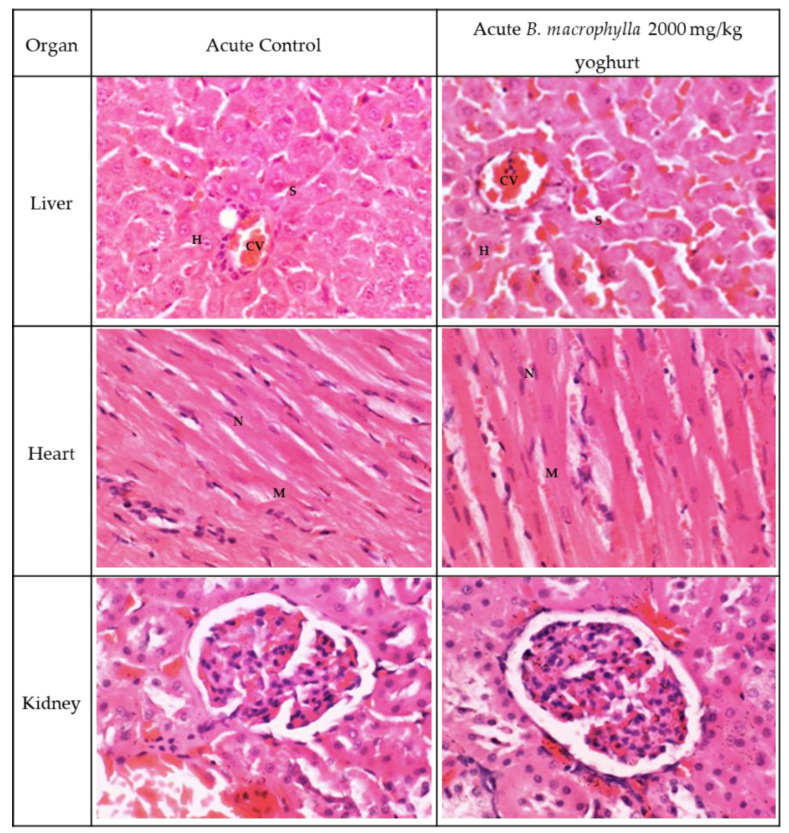
A section on histology was included in the acute toxicity study. There is no evidence of an abnormal lesion or tubular dilation in the kidney histology. It appears that the renal corpuscle is normal. In the kidney histology section, there were no abnormalities found at all. The histology of the heart section reveals that the architecture of the heart is normal with no evidence of inflammation. The myocardium is denoted by M and the nucleus by N. Standard strands of hepatocytes (H), sinusoids (S), and the central vein (CV) are all visible in the section of the liver. Since there was no evidence of steatosis, lobular inflammation, or hepatocyte ballooning, the liver was given a score of 0 on the grading scale.

**Figure 2:**
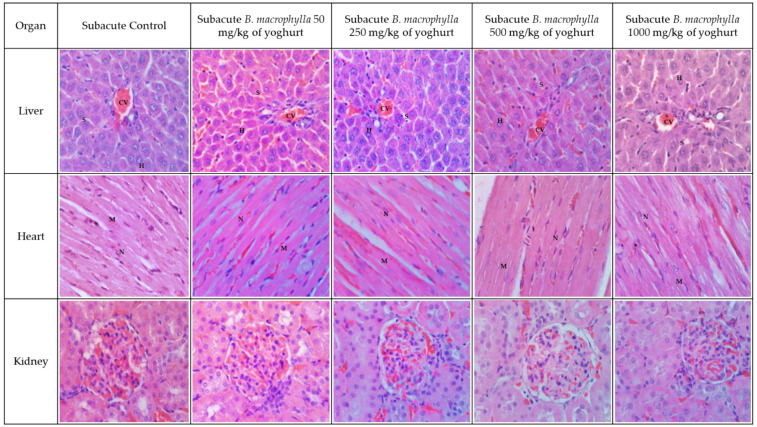
Kidney, liver, and heart histology for subacute toxicity in the subacute control (SC) group and the BMY supplementation groups at varying doses (BMY 50, BMY 250, BMY 500, BMY 1000). There is no abnormal lesion or tubular dilation in the kidney’s histology. The renal corpuscle appears normal. The kidney histology section showed no abnormalities. Histological examination of a heart tissue sample revealed no evidence of inflammation or disruption to the heart’s normal architecture. The myocardium is denoted by M and nucleus by N. Hepatocytes (H), sinuses (S), and the central vein (CV) can all be seen in a typical liver section.

The oral administration of a dose of BMY equal to 2000 mg/kg in SD rats did not result in any mortalities in the treated rats. The animals all behaved normally through the research project, and they all made it to the end of the 14-day experiment period alive. During the observation period, there were no distinguished clinical shifts that they exhibited. In terms of body weight, there was no noticeable difference between the control group and the group that received 2000 mg/kg BMY (Table 4). However, after oral administration of BMY, all of the animals showed a normal increase in body weight without a significant difference between the control group and the treated group. This was the case even though both groups had been given BMY. Based on relative organ weight, there was found to be no significant difference between the two groups (Table 5). Analyses involving haematological and serum biochemistry revealed that BMY did not result in any significant changes in any of the parameters (Table 6 and Table 7), demonstrating that it remains within the reference range. The histopathological examination of the liver and kidney in both the control group and the group that was treated with 2000 mg/kg BMY revealed no abnormalities, as can be seen in Figure 1 and Figure 2. As a direct consequence of this, the LD50 value for BMY was found to be greater than 2000 mg/kg of body weight.

### 2.5. Subacute Toxicity Assessment

#### 2.5.1. Body Weight, Food and Water Intake

Table 8 presents the results of measurements that were taken to determine the average body weight of rats that were part of the subacute^1^ and subacute satellite^2^ groups. It was found that neither the subacute nor the subacute satellite group experienced any significant shifts in their body weight. Although there was no significant difference between the groups of animals after the oral administration of BMY, all of the animals showed a normal increase in their body weight. There was also no significant difference found in caloric intake, total food consumption, or water consumption for the sub-acute group that received repeated doses of SC, BMY 50, BMY 250, and BMY 1000 over the course of 28 days. Moreover, during the 42 days of subacute satellite groups (SSC and SST 1000), there was no significant difference observed in the rats’ calorie intake, total food consumption, or water intake (Table 9).

#### 2.5.2. Relative Organ Weight

Table 10 displays the results of the organ relative weight measurements taken from SD rats that were assigned to the subacute^1^ or subacute satellite^2^ groups. The relative weight of the kidneys, liver, lungs, and heart shows that there were no significant differences (*p* < 0.05).

#### 2.5.3. Haematology and Biochemistry Analysis

According to Table 11, the haematology parameters of the subacute groups^1^ differ significantly (*p* < 0.05) from one another, except for MCH, MCHC, and lymphocytes. Significant differences are detected in MCH value between rats from groups BMY50 and BMY1000 compared to the SC group. MCHC value also shows a significant increase in all groups supplemented with various doses of BMY compared to the SC group. Lymphocytes’ level also displays a significant decrement in BMY 50 and BMY 500 groups compared to the SC group. In the subacute satellite groups, on the other hand, no significant changes were seen in any of the haematology parameters. Table 12 presents the serum biochemical parameters that have been categorised as either renal parameters (such as sodium, potassium, chloride, urea, and creatinine) or liver parameters (such as total protein, albumin, globulin, alanine aminotransferase (ALT), aspartate aminotransferase (AST), and alkaline phosphatase (ALP)). Renal parameters for subacute groups^1^ show no significant changes. In contrast, the liver parameters of the subacute groups^1^ display a significant increase in the albumin level of both the BMY50 and BMY500 groups in comparison to the SC group. When compared to the SC group, the levels of globulin are found to be significantly decreased in the BMY 500 and BMY 1000 groups. The remaining liver parameters for the subacute group exhibited no significant difference. However, in subacute satellite groups, all renal and liver parameters show no significant changes. 

#### 2.5.4. Histopathological Changes

**Figure 3:**
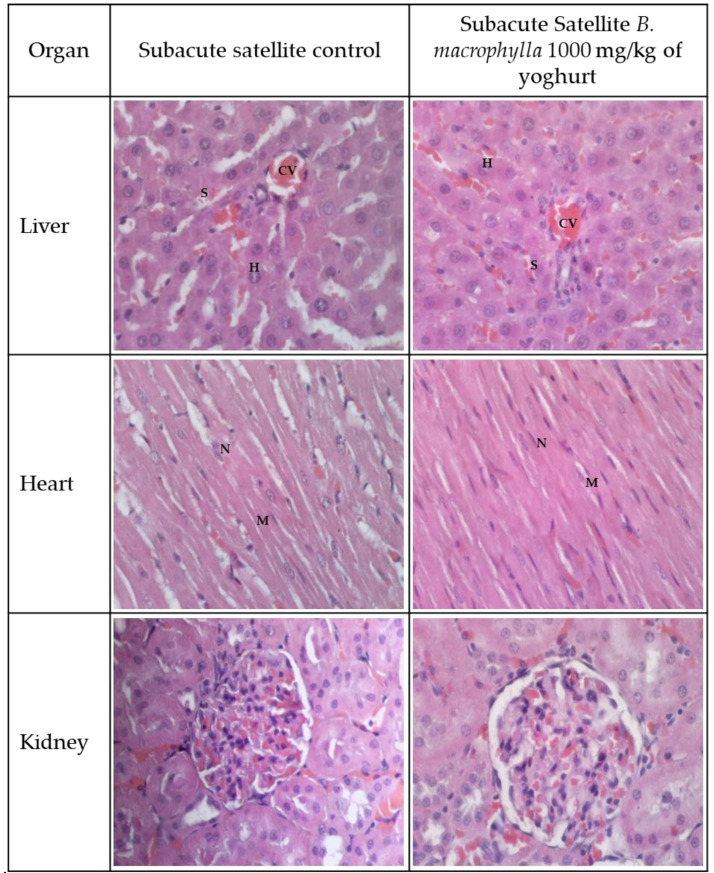
Histology of liver, kidney and heart for subacute satellite control (SSC) and subacute satellite 1000 mg/kg (SST 1000). There is no abnormal lesion or tubular dilation in the kidney’s histology. The kidneys appear to have healthy corpuscles. The kidney histology section showed no abnormalities. Histological examination of a heart tissue sample revealed no evidence of inflammation or disruption to the heart’s normal architecture. The myocardium is denoted by M and nucleus by N. Hepatocytes (H), sinuses (S), and the central vein (CV) can all be seen in a typical liver section.

Figure 2 and Figure 3 illustrate the histological findings that were discovered in the kidney, liver, and heart sections of the subacute and subacute satellite groups, respectively. Compared with the SC group, the histopathological images of the kidney sections of rats supplemented with BMY 50, BMY 250, BMY 500, and BMY 1000 showed no abnormalities. Rats that were given BMY had a structure that was comparable to that of rats in group SC in terms of their glomeruli, tubules, and interstitial space. In all of the groups that were given BMY, there were no indications of inflammation, pyknotic nucleus, or congestion of white blood cells that could be seen. In the subacute satellite group for subacute toxicity, the SST 1000 showed kidney architecture that was comparable to that of the SSC group. There was no inflammation, cell congestion, or pyknotic nucleus in either of the groups. Histopathological examination of liver sections taken from the subacute control group as well as groups supplemented with various doses of BMY (50 mg, 250 mg, 500 mg, and 1000 mg) revealed a normal appearance of the central vein and hepatic sinusoids lined by endothelial cells with normal radiating hepatocytes. During the subacute satellite study, the architecture of the SST 1000 liver was found to be comparable with the SSC group. There was no evidence of inflammation or WBC congestion. Histopathology examination of heart tissue obtained from both subacute and subacute satellite groups revealed the presence of transverse striation of cardiac muscle cells, a central nucleus, epicardium, and mesothelial pavement cells.

## 3. Discussion

**Figure 4:**
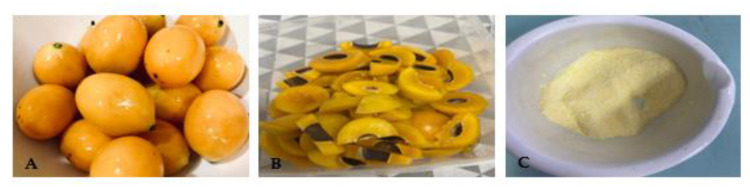
(**A**) Fresh Bouea macrophylla fruit, (**B**) Bouea macrophylla fruit without seed, (**C**) Bouea macrophylla flesh powder.

This study was divided into two phases. In the first phase, ethanol-extracted *Boeau macrophylla* (Figure 4) was incorporated into yoghurt and characterised for its nutritional composition, as well as its phytochemical identification. Based on the nutritional analysis conducted in this study, BMY can be classified under low-sugar yoghurt as it only has 2.72 g of sugar per 100 g. This is according to the cut-off point indicated by European Union (EU) regulations, where a product which contains <5 g of sugar per 100 g can be claimed to be low sugar [7]. Meanwhile, according to the Code of Federal Regulations (CFR) of the Food and Drug Administration of the United States, the composition of fat, protein, and carbohydrate of our formulated BMY meet the standard sets of yoghurt (fat >3.25; protein ≤4.4; carbohydrate ≥7.5) [8].

Based on the phytochemical screening of ethanolic *Boeau macrophylla* conducted in this study, most of the compounds identified belong to the phenolic and terpenoid groups. Across the compound list, the phenolic group with diverse sub-classes were detected and flavonoids were the dominant sub-classes discovered, with 32 compounds out of 66 compounds of the phenolic group. According to the previous study, flavonoids have long to be known to possess anticancer, antioxidant, anti-inflammatory, and antiviral properties [9,10]. Apart from flavonoids, tannins, and phenolic acid are other groups of sub-classes of the phenolic group that are primarily found in ethanolic *Boeau macrophylla.* Both tannins and phenolic acid have been reported to have great antioxidant properties [11]. Specifically, gallic acid, which is common phenolic acid found in this study, was reported to exhibit a nephroprotective effect [12]. In this study, the terpenoid group of compounds is also abundant in ethanolic *Boeau macrophylla,* which possesses various health benefits. For instance, the previous study demonstrated that terpenoids or isoprenoid display antidiabetic action in mice [13] and also exhibit cytotoxicity against tumour cells [14]. 

In the second part of this study project, the formulated BMY was put through its paces in terms of toxicity testing, which included testing for both acute and subacute oral toxicity. BMY can be considered nontoxic based on the acute toxicity classification method. The LD50 of BMY is greater than 2000 mg/kg of body weight, and a single dose of 2000 mg/kg per body weight did not result in any mortality, behavioural abnormalities, postural changes, or significant differences in the rats’ body weights. As shown in the haematology result (Table 6), the WBC was reduced in the 2000 mg/kg but was not significantly different to the control, which might be due to the suppressive effect of some components such as flavonoids and tannins in BMY on bone marrow [15]. There were no perceptible differences found in the relative weight of the organs or the biochemistry of the serum. This was supported by the findings of the histopathological section, which revealed that the kidney, liver, and heart all displayed normal histological characteristics.

Subacute studies provide information on dosage regimens, toxicity to vital organs, and adverse effects that may affect the average life span of an experimental animal. Rats in this study that were subjected to subacute oral toxicity for a period of 28 days showed no signs of mortality, and there were no noticeable changes in their behaviour, regardless of the different doses that were administered (50 mg, 250 mg, 500 mg, and 1000 mg). One of the crucial indications in evaluating an animal’s health status is body weight [16]. In this study, all rats exhibited normal increments of body weight without any significant differences detected. This indicates that BMY did not interfere with the normal metabolism of the experimented rats [17]. The findings of this study also indicate that there were no organ injuries either atrophy or hypertrophy or swell [18,19], since there were no significant differences in relative organ weight between rats supplemented with various doses of BMY and the subacute control group. This indication could be supported by a histopathological evaluation of vital organs that are influenced by metabolic reactions caused by toxicants [20]. Through these histopathological evaluation results, no significant damage was found in the three critical organs (liver, kidney, and heart).

In this present study, haematology parameters were analysed to assess for toxicity in BMY. The haematopoietic system is one of the organs that is most affected by toxic substances and is a good indicator of how healthy or sick a person or animal is [21]. Based on our study, all haematology parameters showed no significant changes except for MCH, MCHC, and lymphocytes. However, the values still lie within the normal reference range [22,23]. Remarkably, the WBCs (Table 11) were slightly reduced across BMY-administrated groups, but were not statistically significantly different to the control group (SC), which might be due to the suppressive effect on growth and differentiation factors in the bone marrow by some components such as flavonoids and tannins in BMY [15]. In satellite, a subacute toxicity study that serves as an adverse event assessment of BMY exposure showed that all haematology parameters did not exert any significant changes between the control group (SSC) and SST 1000. This suggests our formulated BMY did not alter the production of circulating RBC, WBC, or platelets.

In addition to haematology parameters, the utilisation of blood serum or plasma enzymes can function as a diagnostic tool for the detection of organ damage, cell damage, enzyme induction, activation, or inhibition [15]. It is possible to use some different blood parameters to evaluate the extent of the damage done to tissues, potential target organs, and organ function impairment. Renal parameters such as sodium, potassium, chloride, urea, and creatinine, as well as liver parameters such as total protein, albumin, globulin, alanine aminotransferase (ALT), aspartate aminotransferase (AST), and alkaline phosphatase (ALP), are essential in determining whether or not there has been a change in metabolic function involving the kidney and liver [24]. In this study, the subacute administration of BMY did not cause any damage to the kidney, as evidenced by the fact that renal parameters did not significantly change in either the control group or the various BMY-supplemented groups. These insignificant changes in renal parameters were also presented in both satellite subacute groups (SSC and SST 1000). Notably, the level of potassium (Table 12), was slightly increased in the control group (SC) compared across the BMY-administered groups. However, this increment was considered toxicologically irrelevant as this change is not significant statistically. On the other hand, in liver parameters, the level of albumin significantly increased in BMY 50 and BMY 500 groups compared to the SC group. Contrary to this, a significant decrease was detected in the level of globulin in BMY 500 and BMY 1000 compared to the SC group. Yet, the values still lie within the normal reference range [22]. Total protein ALT, AST, and ALP did not exhibit any significant changes across all groups either within subacute groups or within satellite subacute groups. Insignificant changes in ALT, AST, and ALP, as well as creatinine, serve as good indicators of liver and kidney functions [25].

## 4. Conclusions

In conclusion, as BMY produced no evidence of toxicity in both acute and subacute oral toxicity studies, we can conclude that formulated *Bouea macrophylla* yoghurt is non-toxic and tolerable up to 2000 mg/kg. The histology examination revealed no significant changes in the internal organs of the rats, such as the kidney, liver, and heart, in both the control and supplemented groups. To complete the safety profile of this newly formulated BMY, additional studies in future on sub-chronic toxicity, genotoxicity, and compound toxicity are required before proceeding with clinical trials.

## 5. Materials and Methods

### 5.1. Collection of Bouea macrophylla

Fresh fruits of *Bouea macrophylla* were obtained from Kelantan, Malaysia and sent for identification to the Institute of Bioscience, Universiti Putra Malaysia. (UPMSK3154117). 

### 5.2. Extraction Bouea macrophylla Fruit

The flesh of the fruits was separated from the seed and peel. Water was removed from flesh of the fruit by using a fruit juice extractor. Approximately 100 g of ground *Bouea macrophylla* flesh were soaked in 300 mL of food-grade ethanol with a sonicator at 50 Hz with a 1 h interval for 3 days [26]. The mixture was then filtered with Whatman’s filter paper No. 1, and the leftover material was re-extracted twice. The filtrates were combined and then evaporated using a Source Turbo (turbo mode) for 1 h, 5K feet, 40 °C, followed by freeze drying, Labconco, Kansas City, MO. *Bouea macrophylla* flesh powdered ethanol extract was kept at −20 °C.

### 5.3. Preparation of Bouea macrophylla Yoghurt

The incorporation of yoghurt with *Bouea macrophylla* fruit extract was conducted based on Fidelis et al. 2021 [27]. In order to make yoghurt, 100 mL of fresh cow’s milk was brought to a temperature of 72 °C for thirty minutes before being lowered to 45 °C. Approximately, 0.06 g (0.06% *w/w*) of starter culture *(Streptococcus salivarius subsp. thermophilus and Lactobacillus delbrueckii subsp. bulgaricus*) were added. The mixture was incubated using a yoghurt maker at 40–45 °C for 7–10 h until pH dropped to 4.5–4.6 [27]. After that, the yoghurt was stabilised by cooling at 4 °C overnight. 2 g of *Bouea macrophylla* flesh powder extraction was added into 100 mL yoghurt to obtain an approximate final concentration of 2000 mg/100 mL (20 mg/mL). 

### 5.4. Characterisation of Bouea Macrophylla Yoghurt

Nutritional analysis was carried out to determine the protein, energy, fat, sugar and carbohydrate content in the formulated *Bouea macrophylla* yoghurt (BMY). The protein content of the BMY was measured using the Kjeldahl method [28]. BMY yoghurt was placed into a Kjeldahl flask and digested with H_2_SO_4_ and a catalyst. Dilution with H_2_O and neutralisation with sodium thiosulfate to obtain a boric acid solution were then carried out. Then, hydrochloric acid was used to titrate the borate anions, which eventually formed nitrogen. The crude protein was calculated by multiplying with the conversion factor of 6.38 using the formula (1) below:

% N × 6.38 = % protein(1)

The number of carbohydrates was determined by using phenol sulfuric acid, and the results were read off using a spectrophotometer at an absorbance of 490 nm [29]. In order to determine the amount of sugar present in the BMY, the refractometric method was applied. The presence of boundary lines in the refraction field was observed and recorded, and the refractive index was identified [30]. The amount of fat was determined by following the Garber protocol [31], and the amount of energy was determined by following the procedure outlined in the Malaysian food composition database [32]. The Formulas (2 and 3) used to calculate energy (kcal) are as follows:

Energy, kcal/100 g = [Fat] × 9 + [Protein] × 4 + [Carbohydrate] × 4(2)

Energy, kJ/100 g = kcal × 4.184(3)

The pH of the prepared yoghurt was determined using a benchtop pH metre. The microbiological analysis was also performed to determine the numbers of *Lactobacillus* spp., *Bifidobacteria* spp., lactic acid bacteria, and titratable acidity as lactic acid. *Lactobacillus* spp. and *Bifidobacteria* spp. were counted using the pour plate technique and serial dilutions in phosphate-buffer saline (1% PBS). After anaerobic incubation at 37 °C for 72 h, plate counts of *Bifidobacteria* spp. and *Lactobacillus* spp. were performed in Bifidobacterium agar and MRS agar (pH 6.2) containing 1 mg/L sorbitol, respectively. Results were expressed as colony-forming units per mL (CFU/mL) [33]. 

### 5.5. Phytochemical Screening

UHPLC was used to analyse the chemical components (ACQUITY UPLC I-Class system from Water Corp., Milford, MA, United States). Compounds were separated chromatographically using a column manufactured by Waters called ACQUITY UPLC HSS T3 (100 mm × 2.1 mm × 1.8 μm), which was kept at a temperature of 40 °C. Mobile phases A and B were used in a linear binary gradient with water (containing 0.1% formic acid) and acetonitrile (mobile phase B). During the run, the composition of the mobile phase was altered as follows: at 0 min, it contained 1% B; at 0.5 min, it contained 1% B; at 16.00 min, it contained 35% B; at 18.00 min, it contained 100% B; and at 20.00 min, it contained 1% B. The volume of injection was 1 μL, and the flow rate was set to 0.6 mL/min. The UHPLC system was connected to a hybrid mass spectrometer from Waters called a Vion IMS QTOF. This mass spectrometer was fully equipped with a Lock Spray ion source. The ion source was performed in the negative electron-spray ionisation (ESI) mode under the following particular operating conditions: the capillary voltage was 1.50 kV, the reference capillary voltage was 3.00 kV, the source temperature was 120 °C, the desolvation gas temperature was 550 °C, the desolvation gas flow was 800 L/h, and the cone gas flow was 50 L/h. Nitrogen with a purity greater than 99.5% was used for both the desolvation and the cone gas. The data were collected using the high-definition MSE (HDMSE) mode at a scan time resolution of 0.1 s over the range of *m*/*z* 50 to 1500. Consequently, in the course of the run, two separate scans with distinct collision energies (CE) were alternately acquired: a low-energy (LE) scan with a constant CE of 4 eV, and a high-energy (HE) scan in which the CE was ramped from 10 to 40 eV. Both scans were performed in the same manner. The collision-induced dissociation (CID) gas used was argon with a purity of 99.9999 per cent [34].

### 5.6. Experimental Animal

In this particular study, Sprague Dawley rats that had reached the age of five weeks and weighed between 150 and 200 g on average served as the subjects. Acclimatisation of the rats took place over the course of one week at a temperature range of 23–25 °C and a humidity level of 55–60%. The light/dark cycle was 12 h. The procedures that required the use of animals were carried out with the approval of the Animal Care and Use Committee of the Management and Science University (AE-MSU-073, Date of approval: 15th Feb 2017.

### 5.7. Acute Toxicity Study

The acute toxicity experiment was performed according to the protocol described in OECD 407 guidelines. Before the acute toxicity experiment, six male Sprague Dawley rats were split into two groups (*n* = 3). The first group (the controls) received water conveyance. The individuals in Group 2 were given an oral dose of BMY equal to 2000 mg/kg of body weight. After the administration of the substances under study, the rats were monitored at 1, 2, 4, and 6-h intervals for any changes in their general behaviour (including their skin, respiratory activity, tremors, salivation, lethargy, or sleep). Visual observation was carried out to monitor the somatomotor, nervous, circulatory, and respiratory systems. Physical changes, including those to the eyes, fur, skin, and mucous membranes, as well as changes to the behavioural pattern, were also observed. The rats were first observed after 24 h, and then they were observed once a day for the next 14 days. On days 1, 7, and 14, body weight was measured. On day 15, all rats fasted overnight before being anaesthetised and sacrificed. Studies were conducted to determine the LD50 or median lethal dose. Blood and organs were taken for collection.

### 5.8. Subacute Toxicity Study

**Scheme 1:**
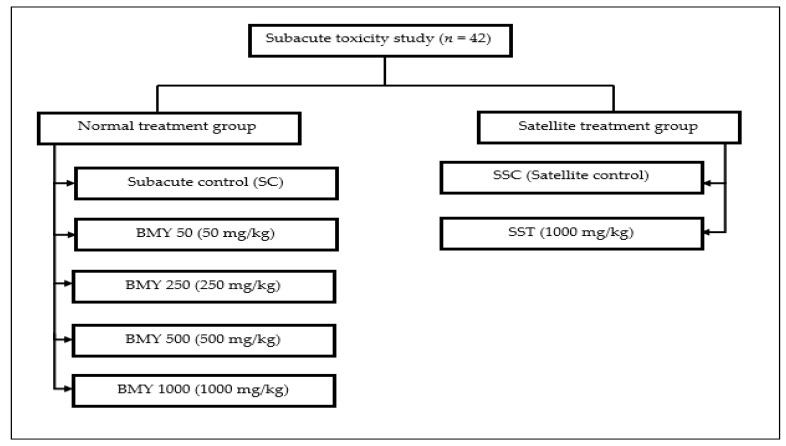
Experimental group of animals in the subacute toxicity study.

A subacute toxicity experiment was performed according to the protocol described in OECD 407 guidelines. In this study, forty-two male rats in good health were used. A normal treatment group and a satellite treatment group comprised the two divisions of this study. In the normal treatment group, rats were put into five groups at random, with six rats in each group (*n* = 6). The groups were designated as subacute control (SC), BMY 50 (50 mg/kg), BMY 250 (250 mg/kg), BMY 500 (500 mg/kg), and BMY 1000 (1000 mg/kg), and were fed BMY orally for 28 days. While the rats were in the satellite treatment group, they were put into two treatment groups at random, with six rats in each group (*n* = 6). The two groups were called SSC (Satellite Control) and SST (1000 mg/kg), respectively. Scheme 1 below illustrates the experimental group of animals. The rats were given supplements by mouth for 28 days (for both normal treatment and satellite treatment groups), and then they had 14 days to rest and be watched without treatment (satellite treatment group only). For the evaluation of toxicokinetics, the satellite group was utilised to determine the level of drug exposure in animals that results in an adverse event [35,36]. The animals were monitored daily for their overall health as well as for any clinical signs of toxicity throughout the duration of the treatments. Variations in body weight were measured on days 0, 7, 14, 21, and 28 of the experiment, respectively. After the experiment, all of the rats were fasted for a full 24 h before being put under anaesthesia so that the cardiac puncture could be performed to collect blood [37]. Blood was collected in both EDTA and plain tubes.

### 5.9. Haematological and Serum Biochemical Analysis

Haematological and biochemical parameters were measured using an automated analyser, Cell Dyn 3700, Abbott Diagnostics, NJ, USA and Cobas C311, Roche Diagnostic, Basel, Switzerland, respectively. For the haematological studies, blood from an EDTA tube was used to measure the haematology parameter, including red blood cell count (RBC), haemoglobin concentration (Hb), haematocrit (HCT), mean corpuscular volume (MCV), mean corpuscular haemoglobin (MCH), mean corpuscular haemoglobin concentration (MCHC), platelets (Pt), and white blood cell count (WBC). For the plain tube, the serum was separated for further analysis of plasma aspartate transaminase (AST), alanine transaminase (ALT), alkaline phosphatase (ALP), total proteins, albumin, total and direct bilirubin, urea, creatinine, and ions (sodium, potassium, and chloride) [37].

### 5.10. Histopathology Analysis

All of the rats were sacrificed by inhaling carbon dioxide immediately following the cardiac puncture. A surgical procedure was performed to remove samples of organs and tissues of interest, such as the liver, kidneys, and heart. These samples were then weighed and examined macroscopically. After determining the relative organ weight and fixing organ samples in formalin, dehydrating them, and embedding them in paraffin, the results were measured. Subsequently, for histological examination, 5 µm thick sections of each sample were stained with hematoxylin/eosin [38,39]. Based on the pathological observations of these tissues, both a macroscopic and a microscopic basis were performed.

### 5.11. Statistical Analysis

The results for body weight, haematological and serum biochemical parameters, as well as relative organ weight, were all expressed as the mean ± SEM. The data were analysed with a one-way ANOVA, and the Tukey HSD Independent T-test was used to compare the results of the two different groups. Software developed by IBM Corp. in New York, NY, USA called IBM SPSS Statistic version 22.0, was used to carry out the statistical analyses.

## Figures and Tables

**Table 1:** Nutritional analysis of BMY.

Parameter	Amount	Unit
Carbohydrate	5.79 ± 0.01	g/100 g
Protein	2.92 ± 0.00	g/100 g
Sugar	2.72 ± 0.00	g/100 g
Fat	3.29 ± 0.01	g/100 g
Energy	64 ± 0.01	kcal/100 g

**Table 2:** Microbiological analysis of BMY.

Microorganism	Number of Colony	Unit
*Lactobacillus* spp.	1.2 × 10^6^	CFU/g
*Bifidobacteria*	2.0 × 10^10^	CFU/g
Titratable acidity as lactic acid	0.599	g/100 g

CFU = colony forming unit; where results are expressed with an “E” this represents an exponential result to a base of 10.

**Table 3:** The phytochemical compound of ethanolic *Boeau macrophylla* under negative ionisation according to different groups of phenolic, terpenoid, alkaloid, benzenoid, carbohydrate, benzopyran, and carboxylic acid.

	Component Name	Formula	Molecular Weight (Da)	Observed Molecular Weight (Da)	Observed *m*/*z*	Mass Error (ppm)	Observed RT (min)
	**PHENOLIC**						
	**Flavonoid**						
1.	2’’-O-Acetylrutin	C_29_H_32_O_17_	652.16395	652.16070	651.15340	−5.0	0.73
2.	5′-Methoxy-bilobetin	C_32_H_22_O_11_	582.11621	582.11600	581.10870	−0.4	0.98
3.	Cryptomerin B	C_32_H_22_O_10_	566.12130	566.12180	565.11450	0.90	0.83
4.	Isolappaol C	C_30_H_34_O_10_	554.21520	554.21330	553.20600	−3.5	6.84
5.	Nevadensin-5-O-β-D-glucoside	C_24_H_26_O_12_	506.14243	506.14210	505.13480	−0.6	12.11
6.	Silybin	C_25_H_22_O_10_	482.12130	482.12330	481.11600	4.10	1.19
7.	6-Methoxykaempferol-3-O-β-D-galactopyranoside	C_22_H_22_O_12_	478.11113	478.11130	477.10410	0.40	12.71
8.	Eriodictyol-7-O-β-D-methyl-glucuronopyranoside	C_22_H_22_O_12_	478.11113	478.11120	477.10400	0.20	8.24
9.	Texifolin-3-O-β-D-glucopyranoside	C_21_H_22_O_12_	466.11113	466.11130	465.10400	0.40	6.62
10.	6-Hydroxykaempferol-3-O-glucoside	C_21_H_20_O_12_	464.09548	464.09570	463.08840	0.50	9.23
11.	Homoplantaginin	C_22_H_22_O_11_	462.11621	462.11580	461.10860	−0.80	12.41
12.	Rengyoside C	C_22_H_32_O_10_	456.19955	456.19940	455.19210	−0.30	13.97
13.	Cinchonain Ⅰb	C_24_H_20_O_9_	452.11073	452.11060	451.10340	−0.20	9.99
14.	Kaempferol-3-O-β-D-glucopyranoside	C_21_H_20_O_11_	448.10056	448.10060	447.09330	0.10	10.29
15.	Sibiricaphenone	C_20_H_28_O_11_	444.16316	444.16280	443.15550	−0.8	7.57
16.	Epicatechin gallate (Epicatechin-3-O-gallate)	C_22_H_18_O_10_	442.09000	442.09000	441.08270	0.00	9.04
17.	5,7,4′-Trihydroxy-8-C-β-D-flavanone glucoside	C_21_H_22_O_10_	434.12130	434.12130	433.11400	0.00	8.70
18.	Quercetin-3-O-α-L-arabinopyranoside	C_20_H_18_O_11_	434.08491	434.08540	433.07810	1.10	9.61
19.	Cnidimonal	C_23_H_16_O_7_	404.08960	404.09140	403.08420	4.50	7.32
20.	Quercetin-3-sulphate	C_15_H_10_O_10_S	381.99947	381.99850	380.99120	−2.70	1.98
21.	Vitamin B2	C_17_H_20_N_4_O_6_	376.13828	376.13800	375.13070	−0.70	6.97
22.	(6aR,11aR)-2,8-Dihydroxy-3,4,9,10-tetramethoxypterocarpan	C_19_H_20_O_8_	376.11582	376.11580	375.10850	0.00	14.26
23.	Protosappanin C	C_16_H_14_O_6_	302.07904	302.07870	301.07140	−1.30	4.74
24.	Quercetin	C_15_H_10_O_7_	302.04265	302.04160	301.03430	−3.40	10.29
25.	Medicagol	C_16_H_8_O_6_	296.03209	296.03180	295.02460	−0.90	0.98
26.	Cnidimol F	C_15_H_14_O_6_	290.07904	290.07880	289.07150	−0.80	5.32
27.	5,7,2’,5’-Tetrahydroxy-flavone	C_15_H_10_O_6_	286.04774	286.04750	285.04030	−0.70	8.70
28.	Genistein	C_15_H_12_O_5_	272.06847	272.06810	271.06090	−1.20	8.70
29.	Astragaline A	C_10_H_11_NO_4_	209.06881	209.06820	208.06090	−2.90	4.69
30.	Nepitrin	C_22_H_22_O_12_	478.11113	478.11130	477.10400	0.30	7.33
31.	2′′-O-Galloylhyperin	C_28_H_24_O_16_	616.10643	616.10540	615.09810	−1.70	10.63
32.	Patuletin-3-O-glucoside	C_22_H_22_O_13_	494.10604	494.10620	493.09890	0.30	6.48
	**Tannin**						
33.	1,2,3,4,6-Penta-O-galloyl-β-D-glucopyranoside	C_41_H_32_O_26_	940.11818	940.12100	939.11380	3.00	9.65
34.	Tellimagrandin Ⅱ	C_41_H_30_O_26_	938.10253	938.10510	937.09780	2.70	9.65
35.	Castalagin	C_41_H_26_O_26_	934.07123	934.07180	933.06450	0.60	9.65
36.	1,2,3,6-Tetra-O-galloyl-β-D-glucopyranoside	C_34_H_28_O_22_	788.10722	788.10900	787.10170	2.30	7.38
37.	Mallotinic acid	C_34_H_26_O_22_	786.09157	786.09320	785.08590	2.10	7.71
38.	Casuariin	C_34_H_24_O_22_	784.07592	784.07720	783.06990	1.60	8.35
39.	Pedunculagin	C_34_H_24_O_22_	784.07592	784.07680	783.06960	1.20	8.57
40.	1,2,6-Tri-O-galloyl-β-D-glucopyranoside	C_27_H_24_O_18_	636.09626	636.09690	635.08960	0.90	6.98
41.	2,4,6-Tri-O-galloyl-β-D-glucose	C_27_H_24_O_18_	636.09626	636.09690	635.08960	1.00	7.19
42.	3,6-Di-O-Galloyl-β-D-glucose	C_20_H_20_O_14_	484.08531	484.08560	483.07830	0.50	5.38
43.	Glucosyringic acid	C_15_H_20_O_10_	360.10565	360.10530	359.09800	−0.90	6.31
44.	Ellagic acid	C_14_H_6_O_8_	302.00627	302.00610	300.99880	−0.60	8.88
	**Phenylpropanoid**						
45.	Tubuloside A	C_37_H_48_O_21_	828.26881	828.27250	827.26520	4.50	0.88
46.	Suffruticosol A	C_42_H_32_O_9_	680.20463	680.20340	679.19610	−1.90	0.56
	**Phenolic acid**						
47.	1-Galloyl-glucose	C_13_H_16_O_10_	332.07435	332.07450	331.06720	0.50	1.78
48.	3-Ethoxy-4,5-dihydroxybenzoic acid	C_9_H_10_O_5_	198.05282	198.05230	197.04500	−2.80	7.32
49.	Methyl-3-hydroxy-4-methoxybenzoate	C_9_H_10_O_4_	182.05791	182.05750	181.05020	−2.40	3.16
50.	Gallic acid	C_7_H_6_O_5_	170.02152	170.02110	169.01380	−2.40	1.78
51.	Methyl gallate	C_8_H_8_O_5_	184.03717	184.03670	183.02940	−2.60	4.95
52.	m-Hydroxybenzoic acid	C_7_H_6_O_3_	138.03169	138.03120	137.02390	−3.50	3.58
53.	E-3,4,5-Trimethoxycinnamic acid	C_12_H_14_O_5_	238.08412	238.08380	237.07660	−1.20	7.49
54.	cis-Caffeic acid	C_9_H_8_O_4_	180.04226	180.04180	179.03450	−2.60	5.37
55.	E-p-Coumaric acid	C_9_H_8_O_3_	164.04734	164.04700	163.03970	−2.00	3.91
56.	1-O-Caffeoyl-β-D-glucopyranoside	C_15_H_18_O_9_	342.09508	342.09610	341.08880	2.90	5.37
	**Coumarin**						
57.	Bergaptol-O-β-D-glucopyranoside	C_17_H_16_O_9_	364.07943	364.07920	363.07190	−0.60	7.34
58.	Cichoriin	C_15_H_16_O_9_	340.07943	340.07930	339.07200	−0.40	4.74
59.	Calycanthoside	C_17_H_20_O_10_	384.10565	384.10720	383.09990	4.10	0.57
	**Lignan**						
60.	(-)-Olivil-4’-O-β-D-glucopyranoside	C_26_H_34_O_12_	538.20503	538.20520	537.19790	0.30	8.88
61.	(-)-Secoisolariciresinol-4-O-β-D-glucopyranoside	C_26_H_36_O_11_	524.22576	524.22640	523.21910	1.30	9.83
62.	Indigoticoside A	C_26_H_34_O_11_	522.21011	522.21040	521.20320	0.60	9.02
	**Quinone**						
63.	1,3,6-Trihydroxy-2-methylanthraquinone-3-O-β-D-glucopyranoside	C_21_H_20_O_10_	432.10565	432.10570	431.09840	0.10	11.45
64.	2,5-Dimethoxybenzoquinone	C_8_H_8_O_4_	168.04226	168.04180	167.03450	−2.90	3.46
	**Xanthones**						
65.	1-Hydroxy-2,3,4,5-tetramethoxyxanthone	C_17_H_16_O_7_	332.08960	332.08890	331.08160	−2.10	9.41
66.	Neomangiferin	C_25_H_28_O_16_	584.13773	584.13610	583.12880	−2.80	0.58
	**TERPENOID**						
67.	Terrestrosin I	C_51_H_84_O_25_	1096.53017	1096.5260	1095.51870	−3.80	0.49
68.	Escin Ⅳg	C_53_H_84_O_23_	1088.54034	1088.53660	1087.52930	−3.50	0.49
69.	Quinquenoside Ⅲ	C_50_H_84_O_19_	988.56068	988.55630	987.54900	−4.50	9.62
70.	Yemuoside YM2	C_31_H_42_O_16_	670.24729	670.24750	669.24020	0.30	8.59
71.	Lucidumoside D	C_27_H_36_O_13_	568.21559	568.21650	567.20920	1.60	8.87
72.	Asperuloside	C_18_H_22_O_11_	414.11621	414.11810	413.11080	4.60	0.57
73.	Dihydrobrusatol	C_26_H_34_O_11_	522.21011	522.21030	521.20300	0.30	11.82
74.	Reptoside	C_16_H_24_O_11_	392.13186	392.13170	391.12450	−0.30	4.43
75.	Vogeloside	C_17_H_24_O_10_	388.13695	388.13650	387.12920	−1.20	5.66
76.	Secoxyloganin	C_17_H_24_O_11_	404.13186	404.13150	403.12430	−0.80	2.74
77.	ApocynosideⅠ	C_19_H_30_O_8_	386.19407	386.19380	385.18660	−0.60	6.75
78.	Sweroside	C_16_H_22_O_9_	358.12638	358.12600	357.11870	−1.10	5.07
79.	Gentiopicroside	C_16_H_20_O_9_	356.11073	356.11050	355.10320	−0.70	6.22
80.	Thymol isobutyrate	C_14_H_20_O_2_	220.14633	220.14580	219.13860	−2.20	12.08
81.	Acetylpaeoniflorin	C_25_H_30_O_12_	522.17373	522.17450	521.16720	1.50	11.88
82.	Pseudosantonin	C_15_H_20_O_4_	264.13616	264.13580	263.12850	−1.50	12.08
83.	3,5,7-Trihydroxychromone	C_9_H_6_O_5_	194.02152	194.02160	193.01430	0.30	1.78
	**ALKALOID**						
84.	Dauricumine	C_19_H_24_C_l_NO_6_	397.12922	397.12860	396.12130	−1.50	0.57
85.	Tryptophane	C_11_H_12_N_2_O_2_	204.08988	204.08930	203.08200	−2.90	3.94
86.	Arginine	C_6_H_14_N_4_O_2_	174.11168	174.11120	173.10390	−2.70	0.51
87.	Xanthosine	C_10_H_12_N_4_O_6_	284.07568	284.07640	283.06910	2.50	5.10
88.	Inosine	C_10_H1_2_N_4_O_5_	268.08077	268.08040	267.07310	−1.60	2.02
89.	Guanine	C_5_H_5_N_5_O	151.04941	151.04870	150.04140	−4.70	2.03
90.	Mudanpioside E	C_24_H_30_O_13_	526.16864	526.16620	525.15890	−4.70	5.26
91.	Echinothiophene	C_23_H_26_O_10_S	494.12467	494.12710	493.11980	4.80	2.42
92.	2,3,5,4’-Tetrahydroxystilbene-2-O-β-D-glucopyranoside	C_20_H_22_O_9_	406.12638	406.12640	405.11910	0.00	15.38
93.	Benzyl alcohol xylopyranosyl(1→6)glucopyranoside	C_18_H_26_O_10_	402.15260	402.15230	401.14500	−0.70	6.53
94.	Cnideoside B	C_18_H_22_O_10_	398.12130	398.12330	397.11600	4.90	0.57
95.	Isomaltose	C_12_H_22_O_11_	342.11621	342.11620	341.10890	−0.10	0.69
96.	Koaburaside	C_14_H_20_O_9_	332.11073	332.11070	331.10340	−0.10	3.46
	**BENZENOID**						
97.	Sennoside B	C_42_H_38_O_20_	862.19564	862.19810	861.19080	2.80	3.14
98.	Scroneoside B	C_23_H_26_O_11_	478.14751	478.14740	477.14010	−0.30	12.41
99.	2,5-Dihydroxybenzeneacetic acid ethyl ester	C_10_H_12_O_4_	196.07356	196.07300	195.06570	−3.00	5.07
100.	m-Hydroxybenzoic acid	C_7_H_6_O_3_	138.03169	138.03120	137.02390	−3.50	3.58
	**CARBOHYDRATE**						
101.	Mannotriose	C_18_H_32_O_16_	504.16903	504.16970	503.16240	1.30	0.74
102.	Pentose	C_5_H_10_O_5_	150.05282	150.05220	149.04500	−4.00	0.70
103.	Galactose	C_6_H_12_O_6_	180.06339	180.06290	179.05560	−2.60	0.69
	**BENZOPYRAN**						
104.	Norbergenin	C_13_H_14_O_9_	314.06378	314.06440	313.05720	2.10	1.79
	**CARBOXYLIC ACID**						
105.	2-(4-Hydroxybenzyl) malic acid	C_11_H_12_O_6_	240.06339	240.06310	239.05580	−1.40	3.58
106.	2-Hydroxy-1,2,3-propane tricarboxylic acid-2-methyl ester	C_7_H_10_O_7_	206.04265	206.04230	205.03510	−1.50	0.72
107.	Quinic acid	C_7_H_12_O_6_	192.06339	192.06290	191.05570	−2.30	0.65
108.	Shikimic acid	C_7_H_10_O_5_	174.05282	174.05230	173.04510	−2.80	0.65
109.	Phenylpropionic acid	C_9_H_11_NO_2_	165.07898	165.07850	164.07120	−2.90	2.55
110.	Mono-ethyl fumarate	C_6_H_8_O_4_	144.04226	144.04170	143.03440	−3.80	0.69
111.	Methylsuccinic acid	C_5_H_8_O_4_	132.04226	132.04170	131.03440	−4.10	0.69

**Table 4:** Effect of BMY on the body weight (mean ± SEM) of rats at 14 days.

Week/Group	Group	
	Control	2000 mg/kg
1	285.12 ± 10.41	283.52 ± 15.13
2	309.13 ± 10.58	298.82 ± 8.10

*n* = 3. Values are expressed as mean ± standard error of the mean.

**Table 5:** The relative organ weight (mean ± SEM) of Sprague Dawley rats in acute oral toxicity study of BMY.

Parameter/Group	Group	
	Control	2000 mg/kg
Spleen	0.37 ± 0.01	0.48 ± 0.05
Kidney	1.35 ± 0.04	1.33 ± 0.28
Liver	5.75 ± 0.33	6.37 ± 0.34
Lung	1.12 ± 0.10	1.15 ± 0.09
Heart	0.61 ± 0.00	0.66 ± 0.15

*n* = 3. Values are expressed as mean ± standard error of the mean.

**Table 6:** The haematology values (mean ± SEM) of SD rats in acute oral toxicity study of BMY.

Parameter/Group	Group	
	Control	2000 mg/kg
Hb (g/L)	14.10 ± 1.05	15.73 ± 0.120
RBC (10^12^/L)	7.29 ± 0.57	7.98 ± 0.52
RDW (%)	13.46 ± 0.44	14.06 ± 0.31
PCV (%)	43.33 ± 3.84	47.33 ± 0.33
MCV (fL)	59.33 ± 0.66	59.66 ± 0.86
MCH (pg)	19.33 ± 0.33	19.66 ± 0.33
MCHC (g/dL)	32.66 ± 0.66	33.33 ± 0.33
WBC (10^9^/L)	11.33 ± 2.27	6.50 ± 0.47
Neutrophils (10^9^/L)	3.23 ± 0.83	1.46 ± 0.20
Lymphocytes (10^9^/L)	7.67 ± 1.68	4.67 ± 0.37
Monocytes (10^9^/L)	0.16 ± 0.67	0.16 ± 0.12
Eosinophils (10^9^/L)	0.24 ± 0.20	0.17 ± 0.55
Basophils (10^9^/L)	0.00 ± 0.00	0.00 ± 0.00
Platelet (10^9^/L)	936.67 ± 106.79	1054.33 ± 136.94

*n* = 3. Values are expressed as mean ± standard error of the mean.

**Table 7:** The serum biochemistry values (mean ± SEM) of SD rats in acute oral toxicity study of BMY.

Parameter/Group	Group	
	Control	2000 mg/kg
Sodium (mmol/L)	141.67 ± 0.88	142.00 ± 0.00
Potassium (mmol/L)	6.33 ± 0.17	6.63 ± 0.08
Chloride (mmol/L)	105.33 ± 1.20	105.67 ± 2.33
Urea (mmol/L)	12.03 ± 1.03	8.43 ± 0.79
Creatinine (mmol/L)	29.67 ± 2.02	29.00 ± 1.00
Total protein (g/L)	59.00 ± 2.08	64.00 ± 1.52
Albumin (g/L)	34.67 ± 1.33	38.00 ± 0.57
Globulin (g/L)	24.33 ± 0.88	26.00 ± 1.00
ALP (U/L)	218.3 ± 28.15	225.67 ± 30.20
AST (U/L)	143.00 ± 11.00	136.33 ± 5.23
ALT (U/L)	36.00 ± 3.60	38.67 ± 4.25
GGT(U/L)	13.00 ± 0.00	13.00 ± 0.00

*n* = 3. Values are expressed as mean ± standard error of the mean.

**Table 8:** Weekly body weight changes for subacute toxicity study. ^1^ The group was supplemented with BMY at various doses daily for 28 days in a subacute toxicity study. ^2^ Satellite group for subacute toxicity study which was given water vehicle or BMY at 1000 mg/kg daily for 28 days followed by no treatment for 14 days.

Week/ Group	Subacute ^1^					Subacute Satellite ^2^	
	SC	BMY 50	BMY 250	BMY 500	BMY 1000	SSC	SST 1000
1	250.00 ± 8.56	275.00 ± 11.38	261.83 ± 15.72	272.50 ± 11.74	268.67 ± 14.88	274.83 ± 21.05	263.83 ± 14.98
2	352.50 ± 10.70	330.00 ± 11.03	312.50 ± 14.07	315.00 ± 14.55	320.00 ± 18.66	326.67 ± 18.51	315.83 ± 13.25
3	360.00 ± 10.65	351.67 ± 7.71	351.67 ± 13.27	340.83 ± 13.93	355.00 ± 20.29	364.17 ± 16.60	355.83 ± 15.57
4	361.67 ± 7.03	366.67 ± 10.54	364.17 ± 10.03	360.00 ± 12.97	368.33 ± 21.36	388.33 ± 16.16	387.50 ± 17.74
5	-	-	-	-	-	415.83 ± 17.63	426.67 ± 17.45
6	-	-	-	-	-	428.33 ± 19.78	431.67 ± 17.78

*n* = 6. Values are expressed as mean ± standard error of the mean.

**Table 9:** Total food intake, caloric intake and water intake for both subacute toxicity ^1^ and subacute satellite ^2^.

	Subacute ^1^					Subacute Satellite ^2^	
	SC	BMY 50	BMY 250	BMY 500	BMY 1000	SSC	SST 1000
Total food consumption (g)	737.30 ± 27.50	746.67 ± 15.43	747.83 ± 23.93	735.00 ± 30.31	732.67 ± 51.96	1146.25 ± 29.02	1155.00 ± 35.00
Calorie intake (KJ)	9437.87 ± 353.07	9557.33 ± 197.54	9572.27 ± 306.40	9408.00 ± 224.00	9378.00 ± 665.1	14,672 ± 371.46	14,784.00 ± 448.00
Total water intake (mL)	886.67 ± 11.67	875.00 ± 26.73	880.83 ± 11.67	898.33 ± 21.03	892.50 ± 61.45	1627.50 ± 18.9	1685.83 ± 23.33

*n* = 6. Values are expressed as mean ± standard error of the mean.

**Table 10:** The relative organ weight for both subacute toxicity ^1^ and subacute satellite ^2^.

Parameter/Group	Subacute ^1^					Subacute Satellite ^2^	
	SC	BMY 50	BMY 250	BMY 500	BMY 1000	SSC	SST 1000
Kidney	0.74 ± 0.48	0.82 ± 0.26	0.82 ± 0.24	0.82 ± 0.30	0.81 ± 0.76	0.73 ± 0.31	0.75 ± 0.43
Liver	3.77 ± 0.14	3.95 ± 0.10	3.78 ± 0.10	3.50 ± 0.28	3.63 ± 0.13	3.60 ± 0.18	3.94 ± 0.18
Lung	0.57 ± 0.66	0.65 ± 0.43	0.66 ± 0.49	0.74 ± 0.42	0.74 ± 0.28	0.63 ± 0.02	0.68 ± 0.05
Heart	0.32 ± 0.17	0.37 ± 0.08	0.36 ± 0.15	0.40 ± 0.31	0.39 ± 0.30	0.43 ± 0.07	0.40 ± 0.03

*n* = 6. Values are expressed as mean ± standard error of the mean.

**Table 11:** The haematology values for both subacute toxicity ^1^ and subacute satellite ^2^.

Parameter/Group	Subacute ^1^					Subacute Satellite ^2^	
	SC	BMY 50	BMY 250	BMY 500	BMY 1000	SSC	SST 1000
Hb (g/L)	15.53 ± 0.56	16.22 ± 0.39	17.10 ± 0.24	16.12 ± 0.37	17.00 ± 0.39	16.9 ± 0.29	16.7 ± 0.58
RBC (10^12^/L)	8.85 ± 0.28	8.43 ± 0.25	8.95 ± 0.20	8.55 ± 0.17	8.77 ± 0.14	9.12 ± 0.28	9.17 ± 0.38
RDW (%)	17.65 ± 0.43	16.53 ± 0.49	16.70 ± 0.38	16.48 ± 0.56	16.38 ± 0.25	16.2 ± 0.68	16.9 ± 0.51
PCV (%)	57.33 ± 1.48	55.50 ± 1.57	58.50 ± 0.89	54.50 ± 1.26	56.17 ± 1.25	55.00 ± 0.89	53.9 ± 1.99
MCV (fL)	62.67 ± 0.71	66.00 ± 0.58	65.33 ± 0.56	63.67 ± 0.49	64.00 ± 1.10	60.7 ± 0.98	58.70 ± 0.5
MCH (pg)	18.00 ± 0.31	19.33 ± 0.21 *	18.83 ± 0.17	19.00 ± 0.00	19.20 ± 0.37 *	18.00 ± 0.70	18.30 ± 0.21
MCHC (g/dL)	28.20 ± 0.20	29.33 ± 0.21 *	29.33 ± 0.21 *	30.00 ± 0.00 *	30.40 ± 0.24 *	30.83 ± 0.30	31.20 ± 0.17
WBC (10^9^/L)	21.28 ± 2.45	12.17 ± 1.95	14.37 ± 1.66	10.53 ± 1.79	14.85 ± 0.89	11.43 ± 2.05	17.10 ± 4.76
Neutrophils (10^9^/L)	3.10 ± 0.48	2.78 ± 0.92	2.67 ± 0.48	3.85 ± 1.57	2.47 ± 0.21	2.81 ± 0.28	2.30 ± 0.38
Lymphocytes (10^9^/L)	13.57 ± 0.61	8.23 ± 1.42 *	10.60 ± 1.24	7.98 ± 1.10 *	11.70 ± 0.76	7.40 ± 1.50	7.70 ± 1.83
Monocytes (10^9^/L)	0.67 ± 0.10	0.90 ± 0.21	0.88 ± 0.12	1.24 ± 0.40	0.50 ± 0.03	0.52 ± 0.11	0.86 ± 0.18
Eosinophils (10^9^/L)	0.20 ± 0.02	0.16 ± 0.02	0.20 ± 0.03	0.15 ± 0.03	0.14 ± 0.01	0.16 ± 0.04	0.17 ± 0.07
Basophils (10^9^/L)	0.13 ± 0.02	0.05 ± 0.02	0.05 ± 0.02	164.35 ± 164.33	0.12 ± 0.08	0.05 ± 0.02	0.02 ± 0.02
Platelet (10^9^/L)	1036.83 ± 35.07	1115.00 ± 52.08	1084.33 ± 86.32	854.17 ± 164.97	1227.00 ± 56.19	982.90 ± 49.2	1060.90 ± 41.4

*n* = 6. Values are expressed as mean ± standard error of the mean. (*) indicate a significant difference at *p* < 0.05.

**Table 12:** The serum biochemistry values for both subacute toxicity ^1^ and subacute satellite ^2^.

Parameter/Group	Subacute ^1^					Subacute Satellite ^2^	
	SC	BMY 50	BMY 250	BMY 500	BMY 1000	SSC	SST1000
Sodium (mmol/L)	144.50 ± 0.56	144.83 ± 0.40	145.00 ± 0.82	146.00 ± 0.83	143.60 ± 0.40	146.1 ± 0.65	147.5 ± 0.72
Potassium (mmol/L)	9.60 ± 0.25	4.20 ± 1.90	4.35 ± 1.94	6.51 ± 1.40	7.15 ± 1.45	2.87 ± 1.81	3.65 ± 1.66
Chloride (mmol/L)	98.17 ± 0.70	96.67 ± 0.84	98.83 ± 0.40	98.00 ± 0.36	98.33 ± 0.61	99 ± 0.52	101.1 ± 0.48
Urea (mmol/L)	7.02 ± 0.29	9.07 ± 0.68	7.73 ± 0.37	9.43 ± 0.91	8.57 ± 0.59	7.85 ± 0.32	6.35 ± 0.35
Creatinine (mmol/L)	56.00 ± 2.48	54.83 ± 3.73	53.50 ± 1.18	48.00 ± 2.72	46.83 ± 1.25	41.17 ± 7.63	35.00 ± 2.31
Total protein (g/L)	69.67 ± 0.80	70.33 ± 1.45	69.83 ± 0.65	64.33 ± 4.15	67.50 ± 1.67	70.83 ± 1.38	69.33 ± 2.10
Albumin (g/L)	39.80 ± 0.67	42.83 ± 0.70 *	42.67 ± 0.67	42.83 ± 0.60 *	41.40 ± 0.81	40.5 ± 0.99	40.67 ± 0.95
Globulin (g/L)	31.50 ± 1.41	27.50 ± 0.85	27.17 ± 0.91	21.76 ± 4.13 *	25.50 ± 1.02 *	30.33 ± 1.05	28.67 ± 1.23
ALP (U/L)	199.00 ± 8.13	200.50 ± 17.27	175.83 ± 9.36	202.00 ± 9.67	160.00 ± 11.66	147.3 ± 4.94	128.67 ± 7.52
AST (U/L)	118.83 ± 10.33	102.50 ± 8.76	98.33 ± 7.67	103.33 ± 9.49	85.17 ± 3.66	99.83 ± 5.26	82 ± 4.93
ALT (U/L)	64.83 ±4.13	46.83 ± 3.32	53.83 ± 4.18	58.50 ± 5.27	55.330 ± 3.40	52.17 ± 1.25	43.83 ± 1.70
GGT (U/L)	13.00 ± 0.00	13.00 ± 0.00	13.00 ± 0.00	13.00 ± 0.00	13.00 ± 0.00	13.00 ± 0.00	13.00 ± 0.00

*n* = 6. Values are expressed as mean ± standard error of the mean. (*) indicate a significant difference at *p* < 0.05.

## Data Availability

Not applicable.
